# MUTFER2024: A new dataset for South African emotion recognition

**DOI:** 10.1016/j.dib.2025.111592

**Published:** 2025-04-28

**Authors:** Rogerant Tshibangu, Jules R Tapamo

**Affiliations:** a Electrical Engineering Department, Mangosuthu University of Technology, Durban 4031, South Africa; b Discipline of Electrical, Electronic and Computer Engineering, University of Kwazulu-Natal, Durban 4041, South Africa

**Keywords:** Collection methods, Preprocessing techniques, Potential applications

## Abstract

Facial Emotion Recognition (FER) plays a critical role in applications such as human-computer interaction, security, and healthcare. The effectiveness of FER systems largely depends on the quality and diversity of the datasets used for training and evaluation. However, existing FER datasets often lack adequate representation of African populations, leading to racial biases in recognizing emotions across diverse ethnic groups. This issue arises from the predominance of Western-centric datasets used in training FER systems, which results in inaccurate and biased outcomes when applied to African or non-Caucasian faces.

To address this limitation, we introduce MUTFER2024, a novel dataset developed at Mangosuthu University of Technology. MUTFER2024 aims to minimize racial bias in FER systems by providing an extensive collection of facial emotion images from African participants. The dataset comprises 13,032 images collected from 300 individuals, including students and staff members, and is categorized into seven emotion classes: happy, sad, angry, surprised, neutral, disgusted, and fearful.

This paper details the methodology employed in data collection, segmentation, and categorization. Facial emotion images were gathered through structured submission protocols to ensure diversity in expressions. Subsequently, the images were meticulously segmented and categorized into the specified emotion classes. Data were collected under real-world conditions using mobile and computer cameras. The dataset is hosted on GitHub and can be used to train emotion recognition models for underrepresented African populations.

Specifications Table

**Table:** 

Subject	Computer Science
Specific subject area	*Facial Emotion Recognition*
Type of data	Image (JPEG)
Data collection	Images captured via mobile/computer cameras; frontal views; no controlled lighting/hairstyle.
Data source location	Computer Lab FF26 and GF22 in the Department of Electrical Engineering at Mangosuthu University of Technology
Data accessibility	Repository name: Zenodo (GitHub archive) Data identification number (DOI): 10.5281/zenodo.14448590 Direct URL to data: [https://doi.org/10.5281/zenodo.14448590](https://doi.org/10.5281/zenodo.14448590) License: The dataset is publicly available under a CC-BY 4.0 license. Users can download the data directly from the Zenodo DOI link.

## 1 Value of the Data

• MUTFER2024 provides facial emotion images collected from two distinct sources: mobile phone cameras (via a facial emotion game) and computer cameras (via a web app). This dual-source approach ensures variability in image acquisition.
• The dataset includes 13,032 images of seven emotion classes (happy, sad, angry, surprised, neutral, disgusted, fearful) from 300 South African participants, addressing the underrepresentation of African populations in existing FER datasets.
• Images were captured under real-world conditions without constraints on lighting, background, hairstyles, facial hair, or accessories, reflecting natural diversity in facial expressions and environments.
• The dataset supports the training and validation of FER algorithms tailored for African contexts, enabling researchers to benchmark or adapt models (e.g., via transfer learning) for underrepresented demographics.
• Public accessibility on GitHub allows reproducibility and reuse in studies focused on reducing racial bias in emotion recognition systems.

## 2 Background

The MUTFER2024 dataset was created to address the underrepresentation of African faces in existing Facial Emotion Recognition (FER) datasets. Most FER systems are trained on datasets dominated by Western populations, leading to racial bias and reduced accuracy for African demographics [1]. This limitation affects applications in human-computer interaction, healthcare, and security, where inclusive and equitable performance is critical.

MUTFER2024 was developed at Mangosuthu University of Technology to provide facial emotion data from South African participants under real-world conditions. The dataset includes 13,032 images across seven emotion categories, captured without constraints on lighting, backgrounds, or accessories. Such variability aligns with the need for robustness in real-world FER systems, as highlighted in studies on mobile and uncontrolled environments [2,3].

To enhance utility, the dataset was designed to support training under conditions reflective of mobile and embedded systems, where computational efficiency and real-time performance are paramount [4]. Image augmentation techniques (e.g., rotation, blur, gamma contrast) were applied during model training to improve generalization, following methodologies validated in prior FER research [5].

## 3 Data Description

The MUTFER2024 dataset comprises 13,032 facial emotion images collected from 300 South African participants. The images were captured under real-world conditions using mobile phone cameras (via a facial emotion game) and computer cameras (via a web app version of the game).

Technical details of the dataset, including preprocessing steps, augmentation techniques, and participant demographics, are summarized in Table 1. Due to ethical restrictions, sample images are excluded from this manuscript but are accessible in the repository under institutional approval.

**Table 1:** Technical details of the MUTFER2024 dataset.

Category	Details
Image Resolution	48 × 48 pixels (grayscale)
Preprocessing	Face cropping, histogram equalization, resizing
Augmentation	Rotation (±15°), gamma contrast, Gaussian blur, horizontal flip
Participant Demographics	300 participants (62% female, 38% male; ages 18–45)
Emotion Class Distribution	Happy (17.2%), Sad (14.1%), Angry (13.8%), Surprised (14.5%), Neutral (15.5%), Fearful (12.9%), Disgusted (12.0%)

**Dataset Composition**:

• Initial Collection: 16,536 raw images.
• Final Cleaned Dataset: 13,032 images after removing low-quality or irrelevant samples.
• Emotion Categories: Happy (17.2%), Sad (14.1%), Angry (13.8%), Surprised (14.5%), Neutral (15.5%), Fearful (12.9%), Disgusted (12.0%) as shown in Fig. 2.

**Data Collection Process**:

• **Devices**: Images were captured using mobile phone cameras (30% of data) and computer webcams (70%).
• **Conditions**: No constraints on lighting, backgrounds, facial hair, or head coverings. All images feature frontal face views.

**Fig. 1:**
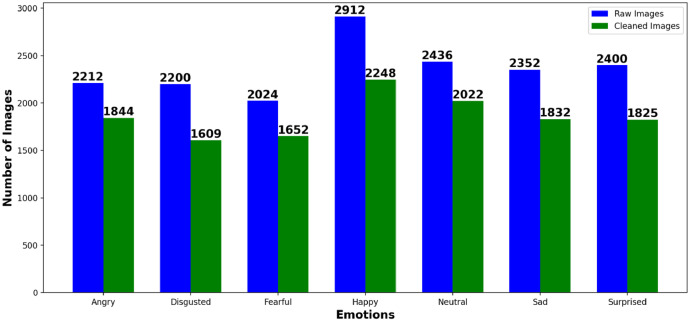
Bar chart comparing raw and cleaned image counts per emotion category.
• **Standardization**: Images were cropped to faces, resized to 48 × 48 pixels, and converted to grayscale.

**Preprocessing**:

• **Cleaning**: Low-quality images (e.g., blurred, occluded) were removed (see Table 2 and Fig. 1).

**Table 2:** Number of images per emotion category before and after cleaning.

Emotion	Raw Images	Cleaned Images
Angry	2212	1844
Disgusted	2200	1609
Fearful	2024	1652
Happy	2912	2248
Neutral	2436	2022
Sad	2352	1832
Surprised	2400	1825
**Total**	**16536**	**13032**

**Augmentation**:

• **Techniques**: Ten methods applied during training, including rotation (±15°), horizontal flip, gamma contrast, and Gaussian blur.
• **Purpose**: To enhance model robustness to real-world variations (e.g., lighting, pose).

**Fig. 2:**
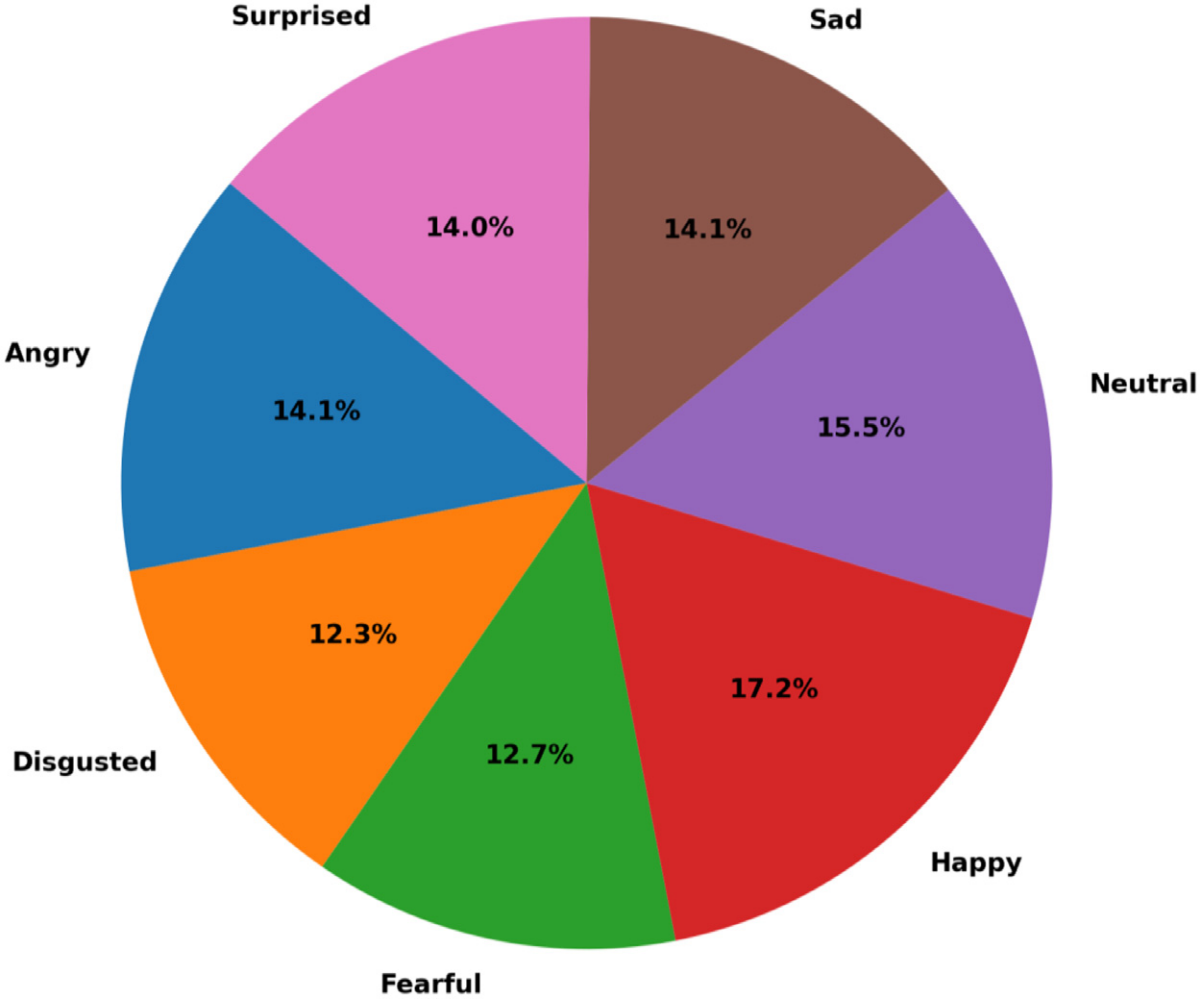
Proportion of emotion categories in the final dataset.
• **Format**: JPEG grayscale images stored in folders labelled by emotion class.

**Dataset Structure**:

• **Class Distribution**: Post-cleaning, emotion categories are relatively balanced (Fig. 2).

## 4 Experimental Design, Materials and Methods

The MUTFER2024 dataset was collected using two main sources: a mobile phone camera (through a facial emotion game) and a computer camera (via a web app version of the same game). The collected data included seven emotional categories: Happy, Sad, Angry, Surprised, Neutral, Fearful, and Disgusted. Images were collected under real-world conditions, with frontal faces, but without strict control over factors such as lighting, background, facial hair, or head coverings. This diversity in the images is crucial for training robust and generalizable facial emotion recognition models, particularly for use in mobile devices, where controlled environments are often unfeasible [5].

### 4.1 Data Collection and Preprocessing

The images collected from mobile and computer cameras varied in size and resolution. To ensure consistency, each image underwent the following preprocessing steps:

1. Face Cropping: Faces were cropped to focus on expressive regions.
2. Resizing: Images were standardized to 48 × 48 pixels, a resolution suitable for convolutional neural networks (CNNs) [6].
3. Grayscale Conversion: Color channels were removed to simplify training and reduce computational costs, as color information is often redundant for facial emotion recognition tasks [7].
4. Illumination Normalization: Histogram equalization was applied to minimize inconsistencies caused by uncontrolled lighting conditions.

These steps ensured uniformity across the dataset, addressing variability from real-world conditions while maintaining compatibility with deep learning frameworks. Resizing and grayscale conversion also align with methodologies for efficient transfer learning, where standardized inputs are critical for fine-tuning pre-trained models [8] Table 3.

**Table 3:** Comparing MUTFER2024 with FER2013, AffectNet, and RAF-DB.

Metric	MUTFER2024	FER-2013	AffectNet	RAF-DB
Size	13,032	35,887	450,000	15,339
Ethnic Focus	South African	Western	Global	Asian
Conditions	Real-world	Controlled	Web-crawled	Lab-controlled
Emotions	7	7	8	7

### 4.2 Image Augmentation and Data Expansion

To address potential data scarcity for training deep learning models, ten image augmentation techniques were applied to the MUTFER2024 dataset. These included rotation (±15°), horizontal flipping, gamma contrast adjustment, Gaussian blur, embossing, and histogram equalization. Augmentation was performed dynamically during model training to improve robustness to real-world variations (e.g., lighting changes, pose diversity) while avoiding overfitting [8].

Augmented images were generated on-the-fly during training and are not included in the publicly released dataset, ensuring ethical compliance and reproducibility. This approach aligns with methodologies validated in prior studies, where dynamic augmentation improves model generalization without inflating dataset size [9].

To further mitigate data limitations, transfer learning was employed using pre-trained models (e.g., ResNet-18). By leveraging features learned from large-scale datasets like ImageNet, transfer learning reduced dependency on the original dataset’s size while enhancing recognition accuracy for African facial expressions [8,9].

### 4.3 Model Training Using Transfer Learning

The augmented dataset was then used for training a convolutional neural network (CNN) for facial emotion recognition. The CNN-based transfer learning method, which uses pre-trained models as starting points, was applied to the MUTFER2024 dataset. As noted in [5], transfer learning allows models to leverage knowledge learned from one task and apply it to a related task, saving both time and computational resources. In this case, pre-trained models, such as VGG or ResNet, were used as the foundation, with the final layers of the model fine-tuned to adapt to the specific task of emotion recognition.

**Fig. 3:**
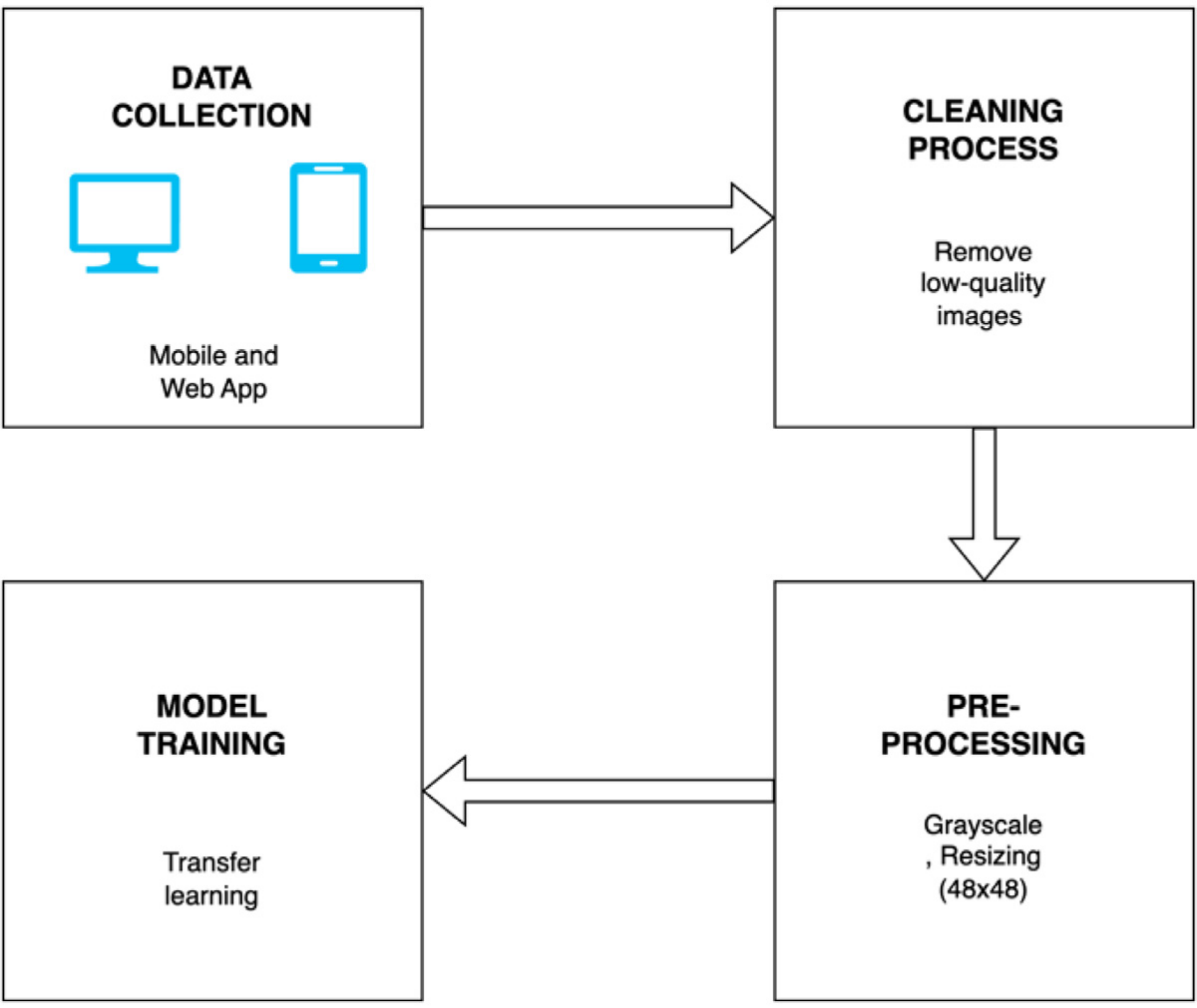
Data collection process.

Transfer learning is particularly effective when there is a limited amount of data, as it helps the model learn better from a smaller dataset by fine-tuning existing features [8]. The use of pre-trained models, as suggested [9], also enables researchers to bypass the long and resource-intensive process of training a model from scratch Fig. 3.

## Limitations


 

• Variability in image quality: The dataset includes images captured under uncontrolled real-world conditions (e.g., inconsistent lighting, background clutter, partial occlusions from facial hair or head coverings) due to the use of mobile and computer cameras.
• Dataset size: While MUTFER2024 contains 13,032 images, smaller than widely used datasets like FER-2013 (35,887 images) or AffectNet (450,000 images). It addresses a critical gap by focusing on underrepresented South African populations under real-world conditions. This targeted diversity enhances its utility for African-centric FER applications, though its smaller scale may limit broader generalizability.
• Geographic specificity: The dataset focuses exclusively on South African participants, prioritizing African representation but potentially limiting utility for non-African contexts. Future work will evaluate cross-regional performance by testing models trained on MUTFER2024 against datasets from other African and non-African regions to assess broader applicability.
• Class imbalance: Despite balancing efforts, Happy (17.2%) and Neutral (15.5%) emotions are overrepresented, while others (e.g., disgusted, fearful) have fewer images post-cleaning. Future versions will employ synthetic minority oversampling (SMOTE) or weighted loss functions to address this uneven distribution.

## Ethics Statement

This study involved human subjects. Informed consent was obtained from all participants prior to data collection. The research was conducted in accordance with the Declaration of Helsinki and received ethical approval from the Mangosuthu University of Technology (MUT) Research Ethics Committee (Protocol Reference: RD1/26/2024). All procedures adhered to the South African National Research Ethics Guidelines (2004) and the MUT Research Ethics Policy.

## CRediT authorship contribution statement

**Rogerant Tshibangu:** Conceptualization, Methodology, Investigation, Data curation, Writing – original draft, Visualization. **Jules R Tapamo:** Supervision, Writing – review & editing.

## Data Availability

Mendeley DataMUTFER2024 (Original data)
Github repositoryFER dataset (Original data)

## Appendix Supplementary materials

Image, application 1

Image, application 2
