# Supplementary material for: MUTFER2024: A new dataset for South African emotion recognition
Source: Data Brief. 2025 Apr 28;60:111592. doi: 10.1016/j.dib.2025.111592 (PMC12136705; doi:10.1016/j.dib.2025.111592)
Supplement: Supplementary file 1 [file mmc1.docx]

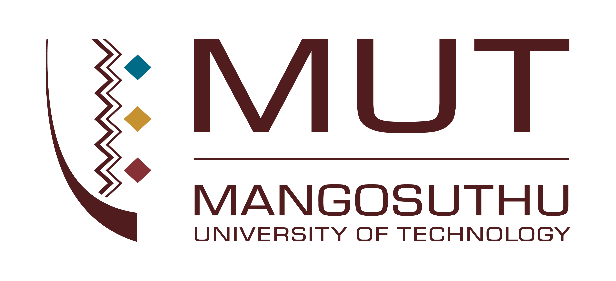


Dear Participant

**Informed Consent Form**

**Project Title:** Exploring Gamified Data Collection and Interpretable Explainability for Enhanced Facial Emotion Recognition

**Study Investigator(s):** Rogerant Tshibangu, Prof. Jules R Tapamo

**Funding Source:** This research project is a requirement of the PhD in Electrical Engineering at Mangosuthu University of Technology (MUT). This project has no external funders.

**Invitation to Participate**

You are invited to participate in the MUTFER2024 image-capturing process as part of a research study. Participation is entirely voluntary, and you may withdraw at any time without negative consequences. This consent form provides information about the study, your role, and any potential risks or benefits. Please read it carefully and feel free to ask any questions.

**What You Will Be Asked to Do**

If you decide to participate, you will be asked to:

- Attend a session where the project will be explained.
- Provide informed consent to participate.
- Use a mobile or web-based application to capture images of your facial expressions, including happiness, sadness, anger, surprise, fear, disgust, and neutral emotions.
- Each session will take approximately 45 minutes.

**Possible Risks and Benefits**

**Risks:** There are no known risks associated with participating in this process. You are free to leave the session or withdraw your participation at any time without consequences.

**Benefits:** Although no direct benefits are guaranteed, your participation will contribute to advancements in facial emotion recognition research, with potential applications in fields like healthcare, education, and human-computer interaction. Additionally, you may gain insight into the research process and associated technology.

**Privacy and Confidentiality**

All collected data will be stored securely in compliance with the Protection of Personal Information Act (POPIA). Your identity will remain confidential, and no personally identifiable information will be published. Data will be stored on secure servers at MUT and accessed only by authorized research team members.

**Reporting of Results**

The results of the study will be prepared in a report for academic purposes and shared with the research community. Participants will be informed of the study’s findings upon completion.

**Withdrawing from the Study**

The results will be compiled into academic reports and shared with the research community. Participants will be notified of the study’s findings upon completion.

**Conflicts of Interest**

None of the researchers have any conflicts of interest in this study.

**Questions and Contact Information**

If you have any questions about your rights as a research participant, you may contact:

- **Researcher:** Rogerant Tshibangu, Email: [Tshibangu.rogerant@mut.ac.za](mailto:Tshibangu.rogerant@mut.ac.za)
- **Supervisor:** Prof. Jules R Tapamo, Email: [tapamoj@ukzn.ac.za](mailto:tapamoj@ukzn.ac.za)
- **Ethics Committee:** Mr Melvin Mothoa, Email: [mothoa.melvin@mut.ac.za](mailto:mothoa.melvin@mut.ac.za)

This research study was reviewed and approved by the Mangosuthu University of Technology Research Ethics Committee (Reference Number: RD1/26/2024).

**Statement of Consent**

By signing below, I confirm that:

- - The study has been explained to me.
  - My questions have been answered.
  - I understand the potential risks and benefits.
  - My personal information will remain confidential.

I also understand that:

- - I have the right to withdraw at any time prior to data analysis without consequences.
  - I can choose not to answer specific questions.
  - I will receive a signed copy of this consent form.

**Consent Options:**

- I consent that I may be quoted and a pseudonym will be assigned to protect my confidentiality: □ Yes □ No
- I consent to keep the identities of other participants confidential: □ Yes □ No
- I consent that the session may be audio recorded: □ Yes □ No
- I consent that the session may be video recorded: □ Yes □ No

**Name:** _____________________________
**Signature:** _____________________________
**Date:** _____________________________
