# Supplementary material for: MUTFER2024: A new dataset for South African emotion recognition
Source: Data Brief. 2025 Apr 28;60:111592. doi: 10.1016/j.dib.2025.111592 (PMC12136705; doi:10.1016/j.dib.2025.111592)
Supplement: Supplementary file 2 [file mmc2.pdf]

DATA USE AGREEMENT

THIS DATA USE AGREEMENT ("Agreement") is entered into as of the date of the last signature by and between Mangosuthu University of Technology, located at Durban, South Africa ("MUT"), and [RECIPIENT ORGANIZATION], located at [ADDRESS] ("Recipient") (each a "Party," and collectively, the "Parties").

WHEREAS, the Parties wish to enter into this Agreement so that MUT may share with Recipient the MUTFER2024 dataset for the purpose of research, public health, or healthcare operations in a manner that complies with applicable laws and regulations.

1. Definitions:
    a) MUTFER2024 Dataset: The data provided to the Recipient under this Agreement. The dataset does not include personally identifiable information or any data that can directly identify an individual.
    b) Individual: The subject of the MUTFER2024 Dataset.

2. Uses and Disclosures: The Recipient agrees to use the MUTFER2024 Dataset solely for the purposes stated in this Agreement and will not use or disclose the data for any other purpose without written consent from MUT.

3. Permissible Use: Recipient warrants that the use and receipt of the MUTFER2024 Dataset will be limited to authorized individuals involved in the specified research or analysis. The Recipient will maintain the dataset's confidentiality and security.

4. Data Access Instructions: Access to the MUTFER2024 Dataset will only be granted upon the completion, signing, and submission of this Data Use Agreement (DUA) by the Recipient. The Recipient must ensure that the completed DUA form is sent to the designated contact at Mangosuthu University of Technology (MUT) for review and approval. Access will not be provided until the DUA has been formally accepted and signed by both Parties.

5. Token Link Reference: Once the Data Use Agreement (DUA) has been duly completed and approved, the Recipient will be provided with a restricted access link (token link) to the MUTFER2024 Dataset. This link will grant access solely to authorized individuals as specified in the DUA. The restricted link is confidential and must not be shared with unauthorized persons. Any misuse of the token link will result in the immediate revocation of access. https://data.mendeley.com/preview/vxtwysdsjw?a=b8103c16-1884-4158-8d9f-5b53c1c13d07

6. Data Ownership: MUT retains ownership of the MUTFER2024 Dataset, including any related information or derivative data created using the dataset.

7. Data Security and Confidentiality: Recipient will:
    a) Not disclose the dataset to unauthorized persons.
    b) Implement safeguards to protect the dataset from unauthorized access or disclosure.

c) Promptly report any breach of data security to MUT.

8. Term and Termination:
    a) Term: This Agreement remains in effect as long as the Recipient has access to the MUTFER2024 Dataset.
    b) Termination: Either Party may terminate this Agreement with ten (10) days' written notice.
    c) Effect of Termination: Upon termination, the Recipient will destroy or return all copies of the MUTFER2024 Dataset.

9. Publication: Recipient shall provide MUT with a draft of any publication or presentation resulting from the use of the MUTFER2024 Dataset at least thirty (30) days prior to submission to any journal or conference.

10. Indemnification: Recipient agrees to indemnify and hold harmless MUT from any claims, damages, or liabilities resulting from the use or misuse of the MUTFER2024 Dataset.

11. Governing Law: This Agreement shall be governed by the laws of the Republic of South Africa.

12. Miscellaneous:

    a) Amendments: Any changes to this Agreement must be in writing and signed by both Parties.
    b) Notices: Any required notices will be given in writing and sent to the addresses listed above.

IN WITNESS WHEREOF, the Parties have executed this Agreement on the dates indicated below.

[RECIPIENT ORGANIZATION]
By: ______________________________
Title: _____________________________
Date: _____________________________

Mangosuthu University of Technology
By: ______________________________
Title: _____________________________
Date: _____________________________
